# Recruitment of HIV-1 envelope occurs subsequent to lipid mixing: a fluorescence microscopic evidence

**DOI:** 10.1186/1742-4690-6-20

**Published:** 2009-03-02

**Authors:** Miao-Ping Chien, Chi-Hui Lin, Ding-Kwo Chang

**Affiliations:** 1 Institute of Chemistry, Academia Sinica, Taipei, Taiwan 11529, ROC

## Abstract

Entry of the human immunodeficiency virus (HIV) into the target cell is initiated by fusion with the cell membrane, mediated through the envelope glycoproteins gp120 and gp41, following engagement to CD4 and the co-receptor. Previous fusion kinetics studies on the HXB2 envelope protein (Env) revealed that Env recruitment occurred at about 13 min concurrent with the lipid mixing. To resolve the temporal sequence of lipid mixing and recruitment, we employed an inhibitory assay monitored by fluorescence microscopy using a gp41 ectodomain (gp41e) fragment, which blocked Env recruitment in stark contrast to the lack of gp41e effect on the lipid mixing. In addition, to demonstrate the mode of action for the inhibition of gp41e, our results strongly suggested that lipid mixing precedes the Env recruitment because lipid mixing can proceed with Env recruitment inhibited by exogeneous gp41e molecules. Importantly, it was found that the random clustering of Env molecules on the membrane surface occurred at ~1 minute whereas the Env recruitment was observed at 13 minutes after the attachment of Env-expressing cell to the target cell. This > 10-fold temporal discrepancy highlights that the productive assembly of Env molecules leading to fusion requires spatio-temporal coordination of several adjacent Env trimers aggregated via directed movement.

## Background

As the first step in the replication cycle of a virus, membrane fusion is mediated by the viral fusion protein. For HIV-1, the fusion protein consists of the non-covalently associated surface subunit gp120 and transmembrane subunit gp41. Attachment of gp120 to the CD4 receptor triggers a cascade of conformational changes in gp120 to expose the co-receptor binding site, which in turn induces structural rearrangement of gp41 and insertion of the fusion peptide into the host cell, forming the pre-hairpin structure. Subsequent refolding of the heptad repeat regions pulls the fusing membranes into close proximity to facilitate the membrane merger.

It has been shown that hemifusion (lipid membrane mixing) is an important intermediate step in the transition to complete membrane fusion for the fusing membranes [1]. Additionally, the assembly of multiple Env proteins on the membrane surface is a critical process in the fusion reaction. Recently, we have examined the function of the co-receptor in HIV-1 HXB2-mediated fusion to dissect kinetically the varying stages of the fusion event [2]. An important finding was that the co-receptor is essential for gp120 shedding from gp41 following CD4 engagement and that the process is gradual, spanning over 10 minutes. It was also found that coreceptor binding accelerates six helix bundles (SHB) formation, promotes hemifusion and complete fusion, and induces the recruitment of adjoining Env subunits to create and sustain the fusion pore. The temporal order of hemifusion and recruitment was, however, not resolved in that study. Together with SHB formation, these three steps constitute the later stages of the fusion event [3]. Because the major portion of energy required for fusion is expected to be expended in these processes (as they involve repulsive hydration force, lipid reconfiguration, pore formation and enlargement, and membrane merger), the rate-limiting step of the fusion likely lies in these steps. Understanding the kinetics of these processes is therefore critical for the mechanistic study of virus-mediated fusion and the pursuit of anti-virus drug and vaccine strategy.

Clustering of the viral fusion proteins on the membrane surface has been previously studied for their possible role in membrane fusion [4,5]. Thus, the distribution of influenza hemagglutinin molecules on the cell surface has been documented at 40 nm resolution, displaying clusters of various size and apparent fluidity [6]. The kinetics of lateral movement and assembly of protein or proteineous complex on the membrane surface has not been elucidated [7]. To resolve the dynamics of lipid mixing and recruitment, we took the competitive inhibition approach in which a recombinant gp41 ectodomain devoid of the fusion peptide segment, termed gp41e, is added to the Env-expressing effector cell in the presence of the target cell at different time points, to observe the effect on the processes being tested. We found that hemifusion was not blocked by the recombinant gp41e protein treatment, whereas Env recruitment was inhibited when the inhibitor was added within 13 minutes of mixing the effector and target cells. The micro-imaging results indicate that hemifusion precedes the Env recruitment and support the view that blocking of Env clustering on the membrane surface is a mechanism of gp41e inhibitory action. In addition, fluorescence recovery after photobleaching (FRAP) was used to demonstrate that the observed functional recruitment dynamics was not a random diffusion, but a highly temporally and spatially orchestrated process. The biological implication of the finding is discussed.

## Results

### Characterization of gp41 ectodomain (gp41e)

A recombinant protein derived from the HIV-1 HXB2 gp41 ectodomain was designed in order to mimic the fusogenic state of viral gp41 fusion protein. Gp41e, comprising 139 residues (gp41 24–154 with 8 His tagged to the C-terminus), includes the 59-residue N-domain, the 28-residue loop region, and the 44-residue C-domain. An 8 His tag was added at the C-terminal end of the gp41e construct to allow for purification by immobilized metal ion affinity chromatography (IMAC). In our study, the complete gp41e, rather than the deletion constructs used in other previous studies [8-11], should better mimic the behavior of HIV-1 gp41. We have shown that gp41e spontaneously folds into an oligomeric, predominantly helical state, and dissolves in distilled water in the native conformation [12].

### Gp41e inhibits content mixing of cell-cell fusion

**Figure 1:**
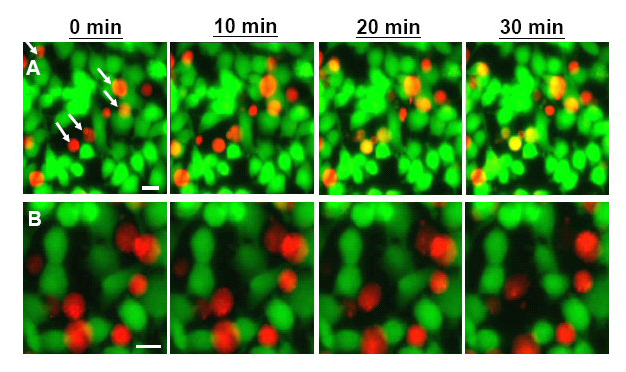
**Content mixing of HIV-1 Env-expressing HeLa cells and CD4-X4-expressing NIH3T3 cells in the absence (A) or presence (B) of gp41e**. For content mixing, HIV-1 Env-expressing HeLa (effector) cells were incubated with CD4-X4-expressing NIH3T3 (target) cells. CMTMR-labeled target cells (red) were co-cultured with calcein-labeled effector cells (green) at 37°C, and dye redistribution was monitored microscopically as described in the Materials and Methods. Content mixing occurred at about 20 minutes after CD4-X4 target cells binding in the absence of gp41e (A, arrow), but not in the presence of gp41e (B). Note that the lower two arrows in panel (A) clearly indicate content mixing while the upper three likely result from overlapping cell images. Scale bar is 10 μm.

Gp41e proteins were tested for the ability to inhibit the fusion of HIV-1 Env-expressing (effector) HeLa cells and CD4-X4-expressing (target) NIH3T3 cells. The target cells were loaded with the cytoplasmic dye 5- and 6-[(4-chloromethyl)benzoyl]amino tetramethylrhodamine (CMTMR, red) for 1 hour at 37°C. CMTMR-labeled target cells were co-cultured with calcein-labeled effector cells at 37°C, and the dye redistribution was monitored microscopically. The aqueous dye mixing was previously determined to occur at about 20 minutes post-incubation [2]. In the present experiment, we added 1 μM of gp41e prior to the co-incubation of effector and target cells. Content mixing was clearly inhibited (Figure 1B), in contrast with the control which was free of gp41e (Figure 1A). The result implies that gp41e prevents the fusion pore formation in the cell fusion cascade. Next, we examined the effect of gp41e on Env recruitment and lipid mixing.

### Addition of gp41e had no effect on the lipid mixing step

**Figure 2:**
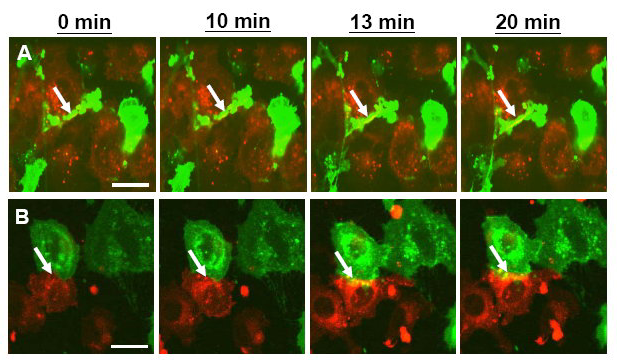
**Lipid mixing of HIV-1 Env-expressing HeLa cells and CD4-X4-expressing NIH3T3 cells in the absence (A) or presence (B) of gp41e**. For lipid mixing, HIV-1 Env-expressing (effector) and CD4-X4-expressing (target) NIH3T3 cells were labeled with the 20 μM DiO (green) and DiI (red) probes, respectively. DiI-labeled target cells were co-cultured with DiO-labeled effector cells at 37°C, and lipid dye mixing was monitored microscopically as described in the Materials and Methods. The effector cells co-cultured with target cells exhibited lipid dye mixing at about 13 minutes upon addition of gp41e (B) or not (A), and the intensity continued to increase up to 20 minutes (arrow). Scale bar is 10 μm.

Complete membrane fusion includes the content mixing and the exchange of lipids between Env-expressing effector cells and target cells [13]. Recently, we have shown that the final step, content mixing, was preceded by Env recruitment and lipid mixing [2]; the temporal order of which was not distinguished, however. Here, we attempted to investigate the influence of gp41e. For this purpose, HXB2 Env-expressing HeLa cells and CD4-X4-expressing NIH3T3 target cells were labeled with two different hydrophobic fluorescent probes, DiO (green) and DiI (red), respectively. DiI-labeled target cells (Figure 2) were co-cultured with DiO-labeled effector cells at 37°C, and lipid dye mixing was recorded in real-time by fluorescence microscopy coupled to a CCD camera. The fluorescent dye mixing results from the exchange of membrane lipids. Target cells co-cultured with effector cells displayed lipid dye redistribution at about 13 minutes, either in the presence or absence of gp41e (Figure 2).

### HIV-1 HXB2 Env recruitment is impeded upon addition of gp41e proteins

**Figure 3:**
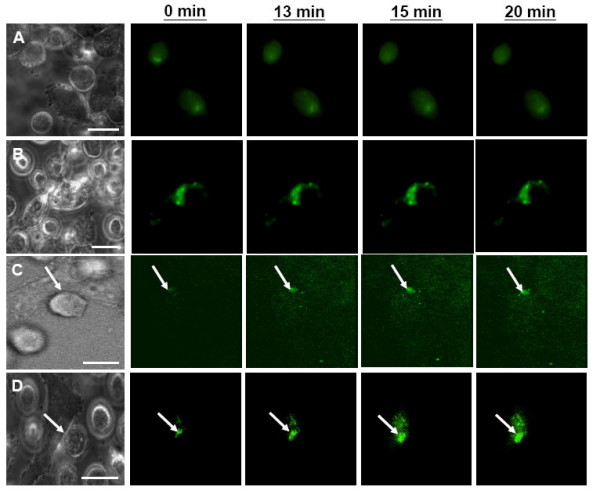
**Recruitment of HIV-1 Env-EGFP (green) on the effector HeLa cells upon engagement of CD4-X4-expressing target NIH3T3 cells in the presence of gp41e added at different time points of 0 (A), 10 (B), 13 (C) and 20 minutes (D)**. Fluorescent images were taken from the same fields of Env-EGFP-expressing HeLa cells (the first bright field) upon addition of target cells. Only images at 0, 13, 15, and 20 minute time points are shown. The movies version is available in Additional Movie 3. After contacting with adherent CD4-X4 cells, HIV-1 Env-EGFP recruitment was observed only at the time points of gp41e added at 13 (C, arrow) and 20 minutes (D, arrow). Scale bar is 10 μm.

**Figure 4:**
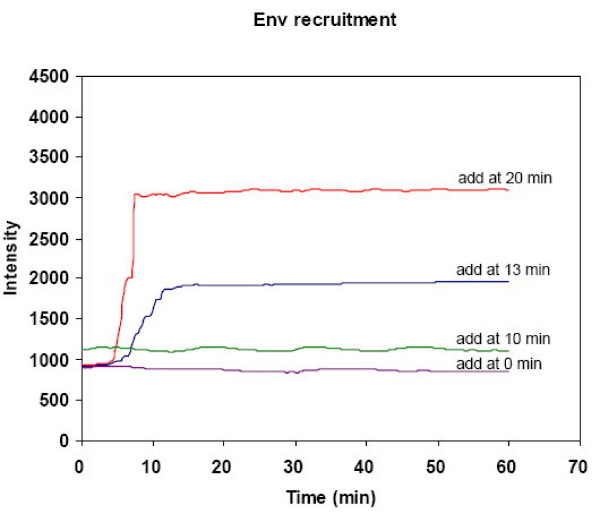
**The intensity representing recruitment of HIV-1 Env-EGFP (green) on the effector HeLa cells from quantification of data from Figure **3. The green fluorescent intensities of HIV-1 Env-EGFP in the presence of gp41e added at different time points of 0, 10, 13 and 20 minutes are shown in the Figure. After contacting with adherent CD4-X4 cells, HIV-1 Env-EGFP recruitment was observed to assay for the effect of adding gp41e at the time points of 13 and 20 minutes.

The higher order structure of multiple Env-receptor complexes is required to form a fusion pore [14,15]. HIV-1 HXB2 Env recruitment could be monitored after binding to target cells expressing CD4-X4 molecules [2]. In addition, HIV-1 Env recruitment was examined by conjugating the EGFP fusion protein (green fluorescence) to Env as a probe for the migration of Env protein on the HeLa effector cells. The recruitment of Env-EGFP on the effector cell surface was recorded by a CCD digital camera upon the addition of target cells. To resolve the dynamics of Env recruitment, we took the competitive inhibition approach in which gp41e is added to the Env-expressing effector cell in the presence of the target cell, at different time points of 0, 10, 13, 20 minutes (Figure 3A–D, respectively) to observe the effect on the processes. Figure 3 shows only 0, 13, 15, 20 minute time points of treatment with gp41e. Initially, the Env-EGFP was distributed on the effector cells without extensive fluorescence clusters found at the contact area with target cells. For gp41e addition at 13 minutes of co-incubation and thereafter (13 and 20 min, Figure 3C–D), onset of the HIV-1 Env-EGFP fluorescence can be observed which clustered at the initial contact site with target cells, and the intensity increased up to 20 minutes post-incubation, at which time the complete membrane fusion (content mixing) occurred. Thus Env-EGFP recruitment was clearly impeded by the gp41e protein added prior to 13 minutes of contact between effector and target cells (Figure 3A–B). Env recruitment in the competitive inhibition approach with gp41e was quantified by the fluorescent tag intensity in Figure 4. The data clearly indicates that, when gp41e was added at 13 minutes after co-incubation of the effector and target cells, the Env recruitment was significantly hindered as compared to the gp41e treatment at the 20 minute time point.

### Recovery time measured by FRAP indicates that Env recruitment is not a lateral diffusion process

**Figure 5:**
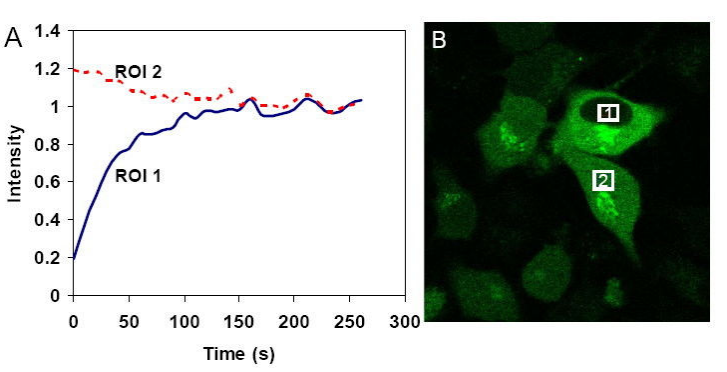
**Fluorescence recovery after photobleaching (FRAP) assay of HIV-1 Env-EGFP**. FRAP was used to analyze the mobility of Env-EGFP, which was measured by analyzing the recovery/return of fluorescent signals into the bleached ROI (region of interest). The fluorescent intensity of ROI 1 (experimental one) decreased to 15% after the pulse and recovered to 65% of its original intensity 1 minute later (Panel A), and the ROI 2 (A) is the control region without bleaching. Panel B displays the fluorescent image of Env-EGFP-expressing effector cells, with ROI 1 of bleaching and ROI 2 of control region indicated.

One intriguing question regarding HIV-1 Env recruitment leading to fusion is whether the process results from the lateral diffusion on the membrane surface. To address the issue, FRAP was used to analyze the mobility of Env in live cells. In this experiment, the laser of a confocal microscope was directed to a specific region of interest in a cell expressing the fluorescent protein to be analyzed. The ROI (region of interest) is exposed to the maximum intensity of the laser, thereby, resulting in prolonged photobleaching of the fluorescent signal (EGFP tagged to Env). The mobility of the tagged protein is then evaluated by the recovery/return time of the fluorescent signal of the bleached ROI. The ROI 2 (Figure 5A) is the control region without bleaching; the fluorescence intensity of which is, therefore, not influenced. Figure 5B shows the fluorescent image of Env-EGFP-expressing effector cells, in which ROI 1 of bleaching and ROI 2 of the control region are indicated. The fluorescence intensity of ROI 1 decreased to 15% immediately after the pulse and recovered to 65% of its original intensity 1 minute later (Figure 5A). Strikingly, the recovery time is an order of magnitude shorter than the time for Env recruitment (~13 min). The disparate kinetics indicates that Env recruitment which leads to the productive fusion event is not a random diffusion process probed by the FRAP measurement. The main distinction for the movement of protein molecules on the membrane surface during the fusogenic process from that in the FRAP experiment lies in the notion that, for the latter, the fusion proteins are not appropriately assembled and thus the movement is not concerted; whereas the movement is highly coordinated spatially and temporally in the former. These points will be further addressed in the Discussion.

## Discussion

### Mechanism of gp41e action on the membrane fusion

The inhibitor approach has been employed to dissect the dynamics of Env-mediated membrane fusion [2,16,17]. Thus, T-20 was used to demonstrate the induction of the pre-hairpin structure by CD4 attachment [16]; anti-HR1 and anti-HR1/HR2 antibodies have been used to differentiate the timing of pre-hairpin exposure and SHB formation at 31.5°C [17]; NC-1 antibody has been used to resolve the temporal order of the pre-hairpin, NC-1 sensitive and SHB formation [2,18]. In the present study, we attempted to use gp41e to dissect the kinetics of hemifusion, gp41 subunit recruitment, and complete fusion. The fluorescence micro-imaging approach adopted effectively circumvented the limitation on the temporal detection of intermediate steps imposed by the complementary function approach – delivery of viral enzyme into target cell – used in the early study of dynamics of virus-induced fusion [19].

Gp41e is the gp41 fragment devoid of the FP (fusion peptide), TMD (transmembrane domain), and CT (cytoplasmic tail) regions; and therefore, it is not anchored to the bilayer core. This property is exploited in the pursuit of a means to resolve the temporal sequence of lipid mixing and recruitment, because it has been shown that the lipid-anchored hemagglutinin mediates hemifusion, but not complete fusion [1], and the recruitment of fusion proteins to promote complete fusion involves spatial and orientational orchestration of several Env trimers. Thus, we reasoned that gp41e, lacking the membrane anchor, should inhibit the process subsequent to recruitment. Similar trimeric recombinant gp41 proteins with entry inhibitory activity have been documented [9].

**Figure 6:**
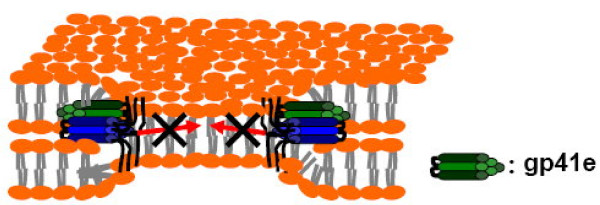
**The mode of action of gp41e inhibition of fusion protein clustering**. The exogenous gp41e trimer (green) interferes with Env recruitment. The resulting Env-gp41e complex blocks the formation of a functional pore and the progress to complete fusion because gp41e lacks a TMD and cytoplasmic tail needed for the disruption of inner leaflets of fusing membranes. This could be a mechanism of fusion inhibition by gp41e.

As expected, gp41e exhibited potent inhibitory activity on the aqueous content mixing between Env-expressing effector cells and CD4-X4-expressing target cells, in agreement with previous studies [9,20]. To pinpoint the step that gp41e interferes, we investigated lipid mixing and the clustering of Env in the late phase of the fusion event. Figure 2 unambiguously illustrates that the lipid dye redistribution occurs at about 13 minutes after co-culture of the effector and target cells, with or without the presence of gp41e. Indeed, the point of gp41e intervention was found at the stage of the fusion protein recruitment as illustrated in Figure 3. The dynamics of gp41e action was further quantified by monitoring the variation of the intensity, representing the extent of clustering of Env, with the time of gp41e addition to the cell mixtures (Figure 4). Remarkably, Env recruitment with gp41e added at 13 minutes (i.e. the starting time of Env recruitment) was somewhat less extensive compared to the inhibitor treatment time of 20 minutes. The result is consistent with the concept that gp41e can block the cell-cell fusion by interfering with Env clustering, as depicted in Figure 6. The dramatic onset of its inhibition at 13 minutes and the observed enhanced effect at 20 minutes of gp41e addition led to the conclusion that the effective Env recruitment does not occur until substantial lipid mixing has taken place.

Based on the mutations on the residues in the NHR-CHR domain of gp41, Markosyan et al. [21] proposed that the inhibitory mechanism of the recombinant core protein rests on the exposure of the CHR region, which binds to the NHR region of the adjacent gp41 molecule, thereby interrupting SHB formation. This latter mechanism can be reconciled with our result that lipid mixing was not blocked by gp41e by considering that lipid mixing may occur during the refolding of the NHR and CHR domains prior to completion of SHB formation. In the previous kinetic study of gp41-mediated fusion [2], the pre-hairpin formation was found to be within a few minutes post-coincubation of effector and target cells. Therefore, gp41e can block the SHB formation at this stage in an open form and later impede fusion protein recruitment at 13 minutes post-coincubation in a trimeric form. Another mode of inhibitory action of gp41 fragments involving the calcium binding site (aa 630–647) [22] is also compatible with the results presented here. Although our inhibition data are most readily explained by the interference of gp41 trimer cluster formation with gp41e adopting a SHB structure, they, nevertheless, cannot identify or distinguish the proposed modes of fusion inhibition by the gp41 ectodomain protein.

To sum up, gp41e significantly inhibited cell-cell content mixing and Env recruitment, but did not inhibit the exchange of lipids.

### Kinetics and functional significance of envelope recruitment for viral fusion

Oligomerization and clustering of the transmembrane proteins of HIV and influenza virus [4,23] have been implicated in the fusion reaction. Thus, previous studies have shown that HIV-1 virions can be recruited to sites of cell contact in the effector dendritic cells [24] and have proposed that contact between the effector and target cells facilitates transmission of HIV-1 by locally concentrating the neighboring virions mediated by Gag and Env proteins [25]. As an extension, we are interested in examining the role of aggregation of the Env molecules in the cell-cell fusion. In our previous study [2], visualization of co-receptor-induced clustering of Env was compatible with the idea that a high order of multiple Env-co-receptor complexes is necessary for fusion pore formation [15]. Interestingly, Env recruitment previously observed at about 13 minutes was concurrent with lipid mixing and continued for a few minutes afterwards [2]. In the present work, we found that gp41e inhibited Env recruitment, but not lipid mixing, implying that lipid mixing is initiated prior to Env recruitment. It is noteworthy that, because both lipid mixing and Env recruitment are progressive (i.e. they last for several minutes), some recruitment occurs before the completion of lipid mixing. In other words, the initial stage of Env clustering takes place while lipid mixing is in progress, or the inception of recruitment of some sites is underway while lipid mixing proceeds near completion at the other sites. This is possible since the inhibition of clustering is negative-dominant (i.e. the aggregation process can be impeded at any time before its completion). The failure of gp41e to block lipid mixing also implies that the step does not require a large number of Env trimeric subunits. In this respect, our finding is not completely disparate with the report that a single functional Env glycoprotein trimer could be adequate to support HIV-1 entry [26,27]. In addition, a gp41 ectodomain construct failed to induce gp120 shedding from the Env-expressing cell alone [9]. The result is in line with the proposal that gp41e exerts its action at the recruitment step which is subsequent to gp120 shedding. according to our previous study [2].

The kinetics of lipid mixing, fusion pore and SHB formation were extensively investigated by Melikyan and coworkers using varied temperature and lipid inhibitors [3,19]. The lag time of ~15 minutes for complete fusion was largely eliminated by subjecting the fusing cells to the temperature arrested stage (TAS, co-incubation of effector and target cells at 23°C for 3 hours; [3]) suggesting that the rate-limiting step is traversed during the stage when the gp41 HR domains was exposed. It was also observed that TAS was concurrent or overlapping with the lipid mixing stage, and SHB did not form until the membrane merger occurred. In the present and previous kinetics [2] studies, the rate-limiting step was deduced at the lipid mixing and recruitment stages, which may lead to pore opening, as explained below. Thus, our results corroborate with that deduced by Melikyan's work.

It was noted that the directional clustering and orientational assembly of several fusion proteins necessary for pore opening and enlargement are associated with large entropy loss and hence unfavorable free energy change; thus, recruitment as a rate-limiting step is energetically reasonable, as exemplified by the flickering of pore opening observed in electric conductance experiments.

Collectively, we hypothesize that the merge of the outer leaflets of apposing membranes initiates with one or a few functional Env trimers at the contact site, and its progress is facilitated by the continuous recruitment of adjacent Env subunits. The spatial and orientational coordination of the clustered Env proteins, along with the FP-TMD interaction to disrupt the inner leaflets around the hemifusion diaphragm, helps drive the fusion reaction to pore formation [26].

The dynamics of protein complexes at the cell surface are determined by the organization, oligomerization state, and interaction with the membrane lipids and other constituents of the cell membrane. Their movement is also directed by the interaction with the cytoskeleton [28], and hence is not totally random.

In the FRAP experiment, the diameter of photobleached ROI is ~5 μm. Using the recovery time of 50 s obtained from Figure 5A, the diffusion coefficient was estimated to be 0.12 μm^2^s^-1^, close to the value 0.09 μm^2^s^-1 ^documented for the influenza hemagglutinin at 22°C on the cell surface [6,29]. If one assumes that the distance of migration for clustering is on the order of < 1 μm, with the diffusion coefficient of 0.12 μm^2^s^-1^, the time taken for an Env subunit to reach the fusion site would be < 1 s, at least two orders of magnitude less than ~10 min observed for Env recruitment here (Figure 3). The discrepancy led to the recognition that recruitment as measured in the present work is a directed organization of several trimeric subunits, whereas the diffusion experiments (Figure 5) measured the random movement of the subunit; conceivably, the proper orientation and juxtaposition of several Env trimers would take longer than the time taken to migrate to the fusion site. Another potentially critical factor is that the FP is inserted into the target cell in our assay, but is free in the FRAP study; the lateral diffusion could be greatly retarded by the double-membrane anchoring of gp41, the effect of which deserves future investigation.

The diffusion rate of oligomers on the membrane surface has been observed to be an order of magnitude smaller than that of a monomer [30]. It was proposed that high order oligomers were trapped in the cytoskeleton mesh and hence had a low rate of hopping between compartments on the membrane, in contrast to monomers or dimers which exhibit free diffusion trajectories and larger diffusion coefficients. The influenza HA molecules have also been found to distribute as elongated clusters, suggestive of arrangement along the cytoskeleton [6]. Hence, the compartmentalization of the cytoskeleton may contribute to the differential dynamics between free lateral diffusion of membrane protein and the recruitment to fulfill fusion function observed here.

The recruitment and assembly of homo- and hetero-oligomers of integral membrane proteins are also essential to the signal transduction process; for instance, activation of G-protein coupled receptors by external ligands triggers the recruitment and hetero-trimeric assembly of G-proteins [31]. Thus the distinction of the directed assembly and diffusion of the membrane proteins addressed in the present work (cf. Figures 3 and 5) may afford some insight into cellular signaling processes.

## Conclusion

### A proposed refined model on the temporal sequence of CD4-co-receptor induced conformational changes of HIV-1 Env protein

**Figure 7:**
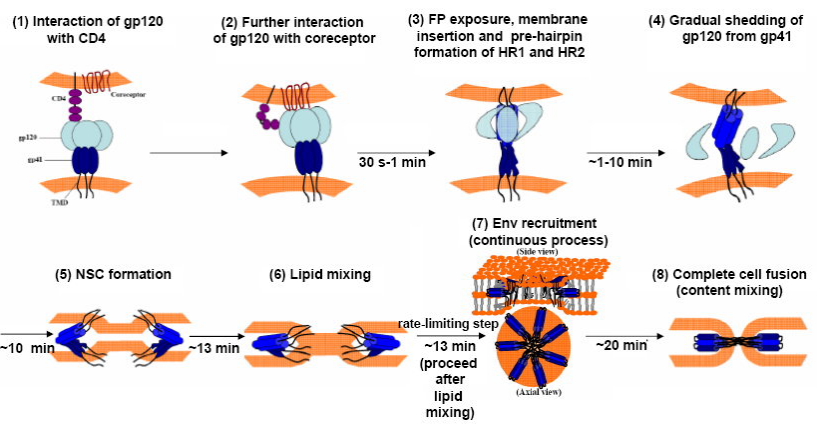
**A schematic illustration of an updated HIV-1 Env-mediated fusion model**. The temporal sequence of Env conformational changes: (1) gp120 interacts with CD4; (2) conformational alteration in both molecules triggers binding to the cognate co-receptor; (3) FP exposure and membrane insertion; pre-hairpin intermediate formation; (4) gradual shedding of gp120 from the gp41 anchor further facilitates refolding of HR1 and HR2 of gp41, leading to (5) formation of NSC – an intermediate bundle structure of gp41 core; (6) mixing of the lipid outer leaflet; (7) recruitment of Env that acts in concert to promote and stabilize fusion pore; (8) full fusion (coalescence of both leaflets of apposing membranes) leading to content mixing.

We have elucidated the functional role of X4 Env in the different stages of the fusion event with emphasis on the kinetic aspect in order to dissect their temporal order [2]. Attachment of the receptor and co-receptor initiates a series of conformational alterations in Env, including extension of FP, insertion of FP into the target membrane, dissociation of oligomeric gp120, gp120 shedding from gp41, refolding of HR1 and HR2, NC-1 sensitive conformation (NSC) formation, Env recruitment, lipid mixing and content mixing. In the present study, we have resolved the temporal order of hemifusion (lipid mixing) and Env recruitment. To recapitulate the present and previous kinetics study, an updated model of the correct temporal sequence of HIV-1 Env conformational changes is depicted in Figure 7. The model could be also applied to the mechanism of other class I fusion protein-mediated membrane fusion.

## Methods

### Cells, plasmids, and antibodies

HXB2 gp160-pSVE7, CD4-pcDNA3, pEGFP-N1 plasmids and their antibodies were gifts from Chen, S. S. (Institute of Biomedical Sciences, Academia Sinica). The HeLa cells, NIH3T3 cells, X4 plasmids (fusin-pcDNA1) and X4 antibodies were obtained through NIH AIDS Research and Reference Reagent Program. The cells were cultured at 37°C in DMEM supplemented with 10% fetal calf serum (GIBCO BRL, Grand Island, NY, USA), penicillin and streptomycin.

### Expression of gp120/gp41, CD4 and X4 on mammalian cells

HXB2 gp120/gp41, CD4 and X4 proteins were expressed on HeLa and NIH3T3 cells (5 × 10^6^), which were transiently transfected using Lipofectamine 2000 (Invitrogen, Carlsbad, CA, USA) by HXB2 gp160-pSVE7 plasmids, CD4-pcDNA3 and X4-pcDNA1 respectively. After 48 hours of expression, cells were subjected to the cell fluorescent image experiments.

### Molecular cloning and protein production of gp41e in bacterial system

How to clone and express the HIV-1 gp41 ectodomain (gp41e) was described in our previous study [12]. In brief, a fragment of 428-base pair was amplified from the plasmid pSVE7'-puro which encodes an ectodomain consisted of the 24th – 154th a.a. of gp41 from the HIV-1 HXB2 strain, and eight histidines at the carboxyl terminus for purification of proteins by the immobilized metal affinity chromatography (IMAC). This cloning was performed with the pETBlue system (Novagen, Merck Co., Madison, WI). The expressed gp41e protein, induced by 1 mM isopropyl-D-1-thiogalactopyranoside, was extracted with the BugBuster protein extraction reagent (Novagen, Merck Co., Madison, WI) according to the manufacturer's protocol, and was then subjected to the IMAC system and purified by HPLC. Molecular mass of the protein was determined by ESI (electron spray ionization)-LC MS (Thermo-Finnigan LCQ series, San Jose, CA).

### Measurements of kinetics of HIV-1 Env recruitment on the cell surface

5 × 10^5 ^HIV-1 Env-EGFP-expressing HeLa (effector) cells were adhered to glass coverslips and placed into an open cell chamber prior to analysis. An equal number of CD4-X4-expressing NIH3T3 (target) cells were diluted in the medium without serum and subjected to the effector cell-coated coverslips in the open cell chamber. For kinetics studies, the cells were imaged on a real-time basis by a MetaMorph microscope (MetaMorph, Carl Zeiss Meditec, Göttingen, Germany), and images were recorded on a CCD digital camera. At least several hundred cells were observed. Image processing was performed using the MetaMorph software (MetaMorph, Carl Zeiss Meditec, Göttingen, Germany).

### Measurements of kinetics of lipid mixing and content mixing

For lipid mixing, HIV-1 Env-expressing (effector) HeLa cells and CD4-X4-expressing (target) NIH3T3 cells were respectively incubated for 15 minutes with 20 μM DiO (green) and DiI (red) in RPMI-1640 medium without serum. The cells were then washed three times with medium or PBS and resuspended at 10^6 ^cells/ml in RPMI-1640 medium without serum. DiI-labeled target cells were co-cultured with DiO-labeled effector cells at 37°C, and lipid dye mixing was monitored in a real-time fashion. For content mixing, the effector and target cells were labeled respectively with calcein AM (green) and CMTMR (red) at concentrations of 10 μM and 20 μM for 1 hour at 37°C. CMTMR-labeled target cells were co-cultured with calcein-labeled effector cells at 37°C, and dye redistribution was monitored in real-time. All the fluorescent images were monitored by fluorescence microscopy (MetaMorph, Carl Zeiss Meditec, Göttingen, Germany) coupled to a CCD camera. The dyes were purchased from Molecular Probes (Eugene, OR, USA). At least several hundred cells were observed. Image processing was performed using MetaMorph software (MetaMorph, Carl Zeiss Meditec, Göttingen, Germany).

### Fluorescence recovery after photobleaching (FRAP) assay

5 × 10^5 ^HIV-1 Env-EGFP-expressing HeLa (effector) cells were adhered to glass coverslips and placed into an open cell chamber prior to analysis. Photobleaching experiments were done using a Zeiss LSM 510 confocal laser-scanning microscope (Carl Zeiss Meditec, Göttingen, Germany). All experiments were done at 37°C. Pixel quantification was performed using the Zeiss LSM software. For the FRAP study, Env-EGFP-expressing HeLa cells were simultaneously imaged, with one region analyzed by FRAP and the other one used as a fluorescence control. The second cell was used as a control, which was still scanned throughout the time course. The fluorescence intensity was monitored simultaneously. Values for the bleached cells were normalized as the percentage of the fluorescence intensity calculated for control cells.

## Abbreviations

HIV: human immunodeficiency virus; Env: envelope; gp41e: gp41 ectodomain; SHB: six helix bundles; FRAP: fluorescence recovery after photobleaching; IMAC: immobilized metal ion affinity chromatography; CMTMR: 5- and 6-[(4-chloromethyl)benzoyl]amino tetramethylrhodamine; HR: heptad repeated; FP: fusion peptide; TMD: transmembrane domain; CT: cytoplasmic tail; NSC: NC-1 sensitive conformation.

## Competing interests

The authors declare that they have no competing interests.

## Authors' contributions

MPC made the fluorescence labeling, FRAP and imaging experiments and initial writing; CHL prepared gp41e constructs; DKC conceived the design and drafted the final version.

## References

[B1] Kemble GW, Danieli T, White JM (1994). Lipid-anchored influenza hemagglutinin promotes hemifusion, not complete fusion. Cell.

[B2] Chien MP, Jiang S, Chang DK (2008). The function of coreceptor as a basis for the kinetic dissection of HIV type 1 envelope protein-mediated cell fusion. Faseb J.

[B3] Cohen FS, Melikyan GB (2004). The energetics of membrane fusion from binding, through hemifusion, pore formation, and pore enlargement. J Membr Biol.

[B4] Ellens H, Bentz J, Mason D, Zhang F, White JM (1990). Fusion of influenza hemagglutinin-expressing fibroblasts with glycophorin-bearing liposomes: role of hemagglutinin surface density. Biochemistry.

[B5] Hernandez LD, Hoffman LR, Wolfsberg TG, White JM (1996). Virus-cell and cell-cell fusion. Annu Rev Cell Dev Biol.

[B6] Hess ST, Gould TJ, Gudheti MV, Maas SA, Mills KD, Zimmerberg J (2007). Dynamic clustered distribution of hemagglutinin resolved at 40 nm in living cell membranes discriminates between raft theories. Proc Natl Acad Sci USA.

[B7] Rath A, Deber CM (2007). Membrane protein assembly patterns reflect selection for non-proliferative structures. FEBS Lett.

[B8] Chan DC, Fass D, Berger JM, Kim PS (1997). Core structure of gp41 from the HIV envelope glycoprotein. Cell.

[B9] Delcroix-Genete D, Quan PL, Roger MG, Hazan U, Nisole S, Rousseau C (2006). Antiviral properties of two trimeric recombinant gp41 proteins. Retrovirology.

[B10] Ji H, Shu W, Burling FT, Jiang S, Lu M (1999). Inhibition of human immunodeficiency virus type 1 infectivity by the gp41 core: role of a conserved hydrophobic cavity in membrane fusion. J Virol.

[B11] Weissenhorn W, Dessen A, Harrison SC, Skehel JJ, Wiley DC (1997). Atomic structure of the ectodomain from HIV-1 gp41. Nature.

[B12] Lin CH, Chang CC, Cheng SF, Chang DK (2008). The application of perfluorooctanoate to investigate trimerization of the human immunodeficiency virus-1 gp41 ectodomain by electrophoresis. Electrophoresis.

[B13] Hart TK, Kirsh R, Ellens H, Sweet RW, Lambert DM, Petteway SR, Leary J, Bugelski PJ (1991). Binding of soluble CD4 proteins to human immunodeficiency virus type 1 and infected cells induces release of envelope glycoprotein gp120. Proc Natl Acad Sci USA.

[B14] Doms RW (2000). Beyond receptor expression: the influence of receptor conformation, density, and affinity in HIV-1 infection. Virology.

[B15] Kuhmann SE, Platt EJ, Kozak SL, Kabat D (2000). Cooperation of multiple CCR5 coreceptors is required for infections by human immunodeficiency virus type 1. J Virol.

[B16] Furuta RA, Wild CT, Weng Y, Weiss CD (1998). Capture of an early fusion-active conformation of HIV-1 gp41. Nat Struct Biol.

[B17] Golding H, Zaitseva M, de Rosny E, King LR, Manischewitz J, Sidorov I, Gorny MK, Zolla-Pazner S, Dimitrov DS, Weiss CD (2002). Dissection of human immunodeficiency virus type 1 entry with neutralizing antibodies to gp41 fusion intermediates. J Virol.

[B18] Jiang S, Lin K, Lu M (1998). A conformation-specific monoclonal antibody reacting with fusion-active gp41 from the human immunodeficiency virus type 1 envelope glycoprotein. J Virol.

[B19] Melikyan GB, Markosyan RM, Hemmati H, Delmedico MK, Lambert DM, Cohen FS (2000). Evidence that the transition of HIV-1 gp41 into a six-helix bundle, not the bundle configuration, induces membrane fusion. J Cell Biol.

[B20] Munoz-Barroso I, Durell S, Sakaguchi K, Appella E, Blumenthal R (1998). Dilation of the human immunodeficiency virus-1 envelope glycoprotein fusion pore revealed by the inhibitory action of a synthetic peptide from gp41. J Cell Biol.

[B21] Markosyan RM, Ma X, Lu M, Cohen CS, Melikyan GB (2002). HIV-1 Env-mediated cell-cell fusion by recombinant cores of gp41 ectodomain. Virology.

[B22] Yu H, Tudor D, Alfsen A, Labrosse B, Clavel F, Bomsel M (2008). Peptide P5 (residues 628–683), comprising the entire membrane proximal region of HIV-1 gp41 and its calcium-binding site, is a potent inhibitor of HIV-1 infection. Retrovirology.

[B23] Danieli T, Pelletier S, Henis Y, White J (1996). Membrane fusion mediated by the influenza virus hemagglutinin requires the concerted action of at least three hemagglutinin trimers. Journal of Cell Biology.

[B24] McDonald D, Wu L, Bohks SM, KewalRamani VN, Unutmaz D, Hope TJ (2003). Recruitment of HIV and its receptors to dendritic cell-T cell junctions. Science.

[B25] Brenchley JM, Schacker TW, Ruff LE, Price DA, Taylor JH, Beilman GJ, Nguyen PL, Khoruts A, Larson M, Haase AT, Douek DC (2004). CD4+ T cell depletion during all stages of HIV disease occurs predominantly in the gastrointestinal tract. J Exp Med.

[B26] Sougrat R, Bartesaghi A, Lifson JD, Bennett AE, Bess JW, Zabransky DJ, Subramaniam S (2007). Electron tomography of the contact between T cells and SIV/HIV-1: implications for viral entry. PLoS Pathog.

[B27] Yang X, Kurteva S, Ren X, Lee S, Sodroski J (2005). Stoichiometry of envelope glycoprotein trimers in the entry of human immunodeficiency virus type 1. J Virol.

[B28] Naghavi MH, Goff SP (2007). Retroviral proteins that interact with the host cell cytoskeleton. Curr Opin Immunol.

[B29] Shvartsman DE, Kotler M, Tall RD, Roth MG, Henis YI (2003). Differently anchored influenza hemagglutinin mutants display distinct interaction dynamics with mutual rafts. J Cell Biol.

[B30] Iino R, Koyama I, Kusumi A (2001). Single molecule imaging of green fluorescent proteins in living cells: E-cadherin forms oligomers on the free cell surface. Biophys J.

[B31] Hamm HE (2001). How activated receptors couple to G proteins. Proc Natl Acad Sci USA.

